# Marine Drugs Acting as Autophagy Modulators

**DOI:** 10.3390/md18010053

**Published:** 2020-01-14

**Authors:** Sergey A. Dyshlovoy, Friedemann Honecker

**Affiliations:** 1 Laboratory of Pharmacology, A.V. Zhirmunsky National Scientific Center of Marine Biology, Far Eastern Branch, Russian Academy of Sciences, Vladivostok 690041, Russia; 2 Department of Oncology, Hematology and Bone Marrow Transplantation with Section Pneumology, Hubertus Wald-Tumorzentrum, University Medical Center Hamburg-Eppendorf, 20251 Hamburg, Germany; 3 Department of Bioorganic Chemistry and Biotechnology, Laboratory of Biologically Active Compounds, School of Natural Sciences, Far Eastern Federal University, Vladivostok 690091, Russia; 4 Martini-Klinik, Prostate Cancer Center, University Hospital Hamburg-Eppendorf, 20251 Hamburg, Germany; 5 Tumor and Breast Center ZeTuP St. Gallen, 9000 St. Gallen, Switzerland

Autophagy (Ancient Greek αὐτόφαγος [*autóphagos*]—“self-devouring”) is defined as a regulated mechanism of the degradation of unnecessary or dysfunctional cellular components [1]. This “self-eating” process may lead to the degradation (selective or non-selective) of organelles and proteins by the lysosome system. Although it can be considered a basic catabolic process, autophagy has only recently become the focus of dedicated research. According to the PubMed database (US National Library of Medicine National Institutes of Health) the number of the annual publications referring to “autophagy” has risen over the last ten years more than 50 times from 128 papers/year (in 2009) to more than 7300 papers/year (in 2019, data for the middle of December 2019) and its total number has reached 46,000 [2]. Moreover, in 2016, the Nobel Prize in Physiology or Medicine was awarded to Prof. Yoshinori Ohsumi for “his discoveries of mechanisms for autophagy” [3]. Autophagy is, therefore, a rapidly and constantly growing field that attracts more and more attention of scientists all over the world. 

Autophagy is implicated in many aspects of human physiology and disease, including cancer, neurodegenerative conditions (Parkinson’s and Alzheimer’s diseases), cardiomyopathy and others, and is essential for the survival and death of mammalian cells [1,3,4]. Interestingly, in pathological conditions, autophagy has been reported to be a double-edged sword. Its activation might be beneficial for the treatment of certain diseases but could turn out to be detrimental for others. Moreover, in conditions like cancer, excessive autophagy may be a positive factor at early stages of tumorigenesis (as it leads to the elimination of cancer cells via type II cell death), but negative at late stages of tumor development (as it might help malignant cells to overcome conditions of cellular stress and activate survival under chemo- and radiotherapy) [5,6]. Therefore, the biochemical tools which allow us to control and manipulate this process are of high impact and interest in biomedical science. Novel small molecules capable of both activation and inhibition of this process are urgently needed.

Over the last few years, the methods of autophagy monitoring have improved significantly. The understanding of the related processes and key molecular players relevant to the different stages of autophagy has led to the development of a variety of tests. These methods allow us not only to distinguish between activators and inhibitors of autophagy (which, interestingly, very often exhibit similar experimental outcomes) but also to identify the autophagic flux stage where the effect of a drug takes place and which molecular target is affected [7]. One of the best and most elaborate guidelines on the assays for autophagy monitoring and its interpretation is published at regular intervals by Daniel Klionsky and colleagues. The last recommendations “Guidelines for the use and interpretation of assays for monitoring autophagy” (3rd edition) was published in 2016 [8], and the newest version is expected to be issued at the beginning of 2020 [9]. For the time being, we recommend the use of most recent recommendations published by Klionsky et al. (Autophagy. 2016;12(1):1-222; PMID: 26799652) [8] as well as by Yoshii and Mizushima (Int J Mol Sci. 2017;18(9):1865; PMID: 28846632) [10].

The more the importance of autophagy is recognized, the more often newly generated compounds are tested for the ability to modulate this physiological process. It seems as if the most exciting and unusual novel structures can be found in marine life forms, as these organisms are by far less well studied compared to terrestrial life forms [11]. Due to the special and often extreme environmental conditions they live in (high pressure, lack of light, salinity, pH), many of these organisms harbor a unique variety of chemical compounds, and there are still many more to be discovered. A good proportion of these compounds exhibit potent biological activity, targeting one or several specific biological processes [12,13]. Not surprisingly, marine natural compounds have come to the attention of scientists searching for new effective autophagy modulators. Accordingly, the number of annually published articles related to “autophagy” and “marine” topics has increased 25 times over the last decade (from 3/year in 2009 up to 76/year in 2019, according to the PubMed database) [14]. The very last review on marine compounds possessing autophagy-modulating activity was published in 2016 by Ruocco et al. [15]. Since then, many new marine-derived substances showing autophagy-modulatory properties have been described and, therefore, are awaiting to be summarized and reviewed.

The current Special Issue “Marine Drugs acting as Autophagy Modulators” of Marine Drugs is a continuation of the Special Issue “Marine Compounds as Modulators of Autophagy and Lysosomal Activity” (Marine Drugs), collecting articles between 2016–2018 [16]. Its aim is to cover the whole scope of agents with autophagy-modulating activity, from novel to previously characterized, including already clinically used marine-derived compounds known to target autophagy both in vitro and in vivo. The issue will present work on compounds that are able to modulate all the different types of autophagy, i.e., macroautophagy, microautophagy, and chaperone-mediated autophagy, with cytotoxic, cytoprotective, pro-survival, or non-cytotoxic biological activity.

We invite you to share with us and all our readers your latest and exciting discoveries!

Dr. Sergey A. Dyshlovoy and Dr. Friedemann Honecker, Guest Editors, Special Issue “Marine Compounds as Modulators of Autophagy and Lysosomal Activity ”, and Editorial Board Members, Marine Drugs.
